# Extracutaneous melanomas: a primer for the radiologist

**DOI:** 10.1007/s13244-015-0427-8

**Published:** 2015-09-03

**Authors:** Abhishek R. Keraliya, Katherine M. Krajewski, Marta Braschi-Amirfarzan, Sree Harsha Tirumani, Atul B. Shinagare, Jyothi P. Jagannathan, Nikhil H. Ramaiya

**Affiliations:** Department of Imaging, Dana Farber Cancer Institute, Harvard Medical School, 450 Brookline Avenue, Boston, MA 02215 and Department of Radiology, Brigham and Women’s Hospital, Harvard Medical School, 75 Francis Street, Boston, MA 02115 USA

**Keywords:** Mucosal melanoma, Ocular melanoma, Immunotherapy, MRI, FDG-PET/CT

## Abstract

**Objective:**

The purpose of this article is to provide a comprehensive review of the imaging features of extracutaneous melanomas.

**Conclusion:**

Extracutaneous melanomas are clinically and biologically distinct from their more common cutaneous counterpart with higher frequency of metastatic disease and poorer overall prognosis. Complete surgical excision is the treatment of choice whenever possible; systemic therapy in the form of conventional chemotherapeutic agents as well as novel targeted agents is used for advanced/ metastatic disease. Multiple imaging modalities including US, CT, MRI and FDG-PET/CT play important roles in the evaluation of the primary tumour, assessment of metastatic disease and monitoring response to treatment. Radiologists should be aware of the typical imaging manifestations of extracutaneous melanoma, the distinct patterns of metastatic involvement as well as treatment response and toxicities associated with newer molecular targeted and immunotherapies to optimally contribute to patient management.

***Teaching points*:**

• *Mucosal melanoma is clinically and biologically distinct from cutaneous melanoma.*

• *Mucosal melanoma has a higher rate of metastatic disease than the cutaneous subtype.*

• *Imaging is helpful in assessment of disease and response to treatment.*

## Introduction

Extracutaneous melanomas are rare, aggressive melanomas that are clinically and biologically distinct from cutaneous melanoma and include mucosal, ocular and leptomeningeal melanomas. According to a study based on data from the North American Association of Central Cancer Registries, extracutaneous melanomas represent only 5 % of all melanoma with approximately 70 % being ocular and rest mucosal subtypes [1]. Extracutaneous melanoma tends to affect older patients and is often detected at an advanced stage, with a worse prognosis than its cutaneous counterpart. Complete surgical excision is the treatment of choice for mucosal melanoma, if feasible; systemic therapy is used for metastatic disease. Knowledge about unique imaging features of extracutaneous melanoma is important in clinical practice because of its rarity and adverse prognosis compared to more common cancers affecting the orbit and various mucosal sites. In this review, we comprehensively describe the epidemiologic, pathophysiologic and clinical features, diagnostic workup, staging and management of extracutaneous melanoma with a focus on the role of imaging. Ocular melanoma, leptomeningeal melanoma and mucosal sites of origin are discussed.

### Differences between cutaneous and mucosal melanoma

Mucosal melanoma is a rare but aggressive form of melanoma that is clinically and biologically distinct from cutaneous melanoma. Compared with cutaneous melanoma, mucosal melanoma has several distinct features. Mucosal melanoma generally occurs in elderly patients, with a median age at diagnosis of 70 years, as opposed to 44 years for cutaneous melanoma. No clear predisposing factors have been identified for mucosal melanoma. Cutaneous melanoma is about 16 times more common among whites than blacks, while mucosal melanoma is only twice as common. Contrary to cutaneous melanoma, which is more common in males, mucosal melanoma is more prevalent in females, with a male-to-female rate ratio of 0.54 [1]. The higher prevalence of mucosal melanoma in the female population is due to the frequency of vulvovaginal melanoma. Approximately 40 % of mucosal melanomas are amelanotic and 20 % are multifocal compared with just 10 % and 5 % for cutaneous melanomas, respectively [2]. Mucosal melanoma is often diagnosed late and is associated with poor outcome, as compared to cutaneous melanoma (5-year overall survival rate of 25 % compared to 81 % with cutaneous melanoma) [2]. Complete surgical excision with clear margins is often difficult to achieve because of the location of these tumours and their multifocal nature.

### Ocular melanoma

#### Epidemiology

Ocular melanoma is the most common primary eye tumour in adults and the second most common type of melanoma after the cutaneous type. The majority (80 %) of the ocular melanomas originate in the uvea (iris, ciliary body and choroid), while the remainder arise in non-uveal sites, including the conjunctiva [1]. The incidence of ocular melanoma increases with age, peaking near the age of 70 years [3].

#### Clinical features and diagnostic workup

Uveal melanoma arises from melanocytes of the iris, ciliary body or the choroid. Choroidal melanoma is the most common subtype of the uveal melanoma comprising 86 % cases [1]. Various host factors that may increase the risk of uveal melanoma include Caucasian race, light skin and eye color, and propensity to sunburn [4]. Also, atypical cutaneous nevi, common cutaneous nevi, cutaneous freckles and iris nevi increase the risk of uveal melanoma [5]. The clinical presentation of uveal melanoma depends on the size and location of the tumour ranging from asymptomatic incidental detection on ophthalmic examination to a variety of visual symptoms. Common clinical features include blurred vision, visual field defect, floaters, pain and increased intraocular pressure. The initial diagnosis of uveal melanoma is usually done on fundoscopic examination. Ocular ultrasound and fluorescein angiography are useful for further characterisation. The tumour node metastasis (TNM) staging of uveal melanoma is done according to the American Joint Committee on Cancer (AJCC) system [6]. In this system, the extent of the primary tumour (T), presence or absence of lymph node involvement (N), and presence or absence of distant metastasis (M) are used for staging the disease. T staging of iris melanoma is done according to tumour extent, associated secondary glaucoma and extraocular ocular extension. Posterior uveal (ciliary body and choroid) melanoma is graded according to tumour basal diameter and thickness, ciliary body involvement and extraocular extension. The regional lymph nodal involvement is classified as NX (cannot be assessed), N0 (lymph node metastasis absent) and N1 (lymph node metastasis present). Similarly, metastatic disease is designated as MX (cannot be assessed), M0 (distant metastasis absent) and M1 (distant metastasis present). Among subtypes of uveal melanoma, iris melanoma is associated with a better prognosis than ciliochoroidal subtype [7]. Primary goals in treating uveal melanoma are preserving vision if feasible and preventing metastatic disease. In the past, enucleation was the standard treatment for uveal melanoma; however, in recent years enucleation has been largely replaced by various forms of radiation therapy (brachytherapy, proton beam irradiation and Gamma Knife) [8].

#### Imaging findings

**Fig. 1:**
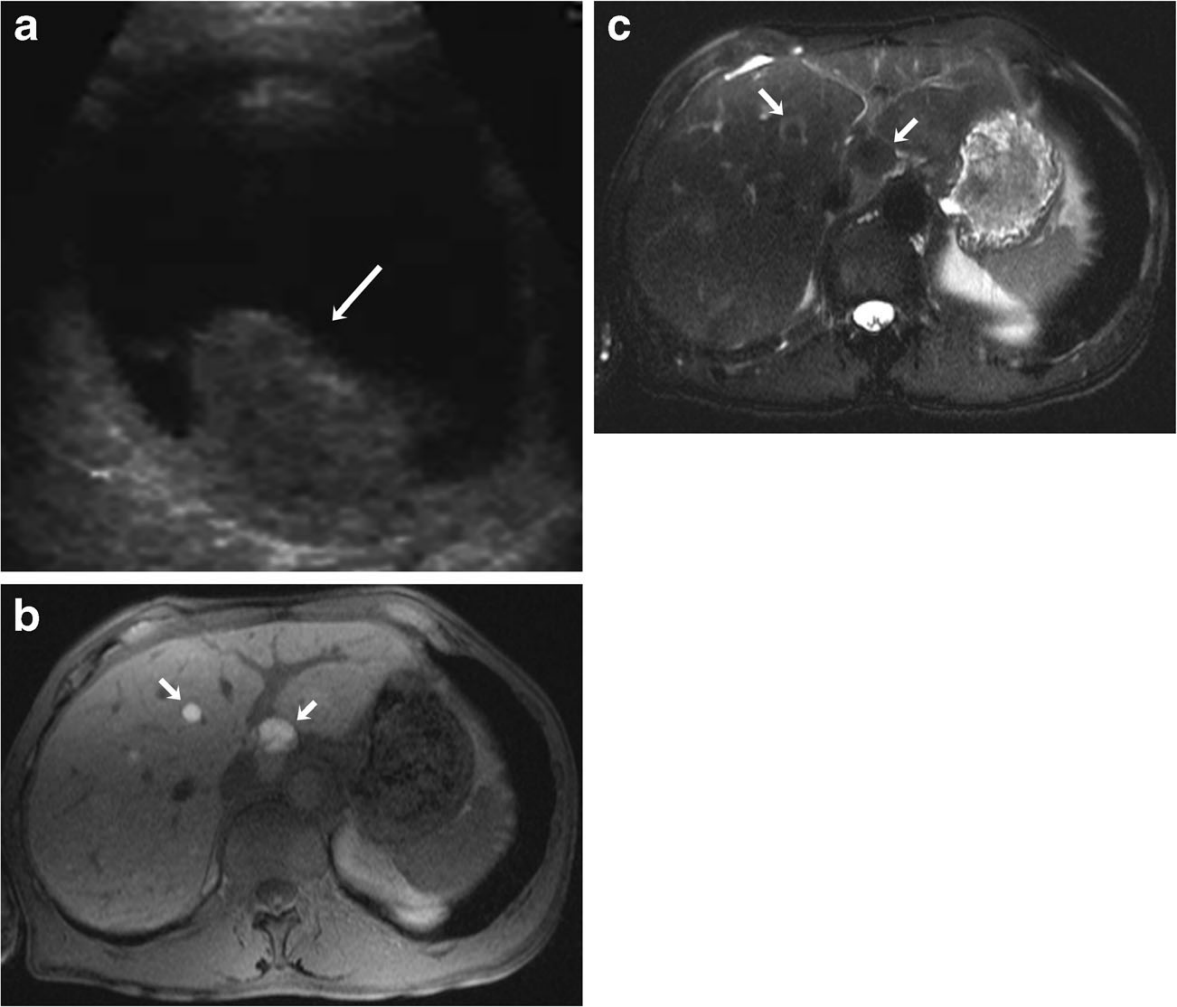
A 52-year-old female with uveal melanoma and hepatic metastases. **a** Grey-scale ultrasound image showing an echogenic mass in the posterior segment of the left globe (arrow). **b** Axial fat-suppressed T1-weighted MR image showing hyperintense hepatic metastatic lesions (arrow). **c** The lesions are hypointense on the corresponding axial fat-suppressed T2-weighted MR image (arrows)

Orbital ultrasound (US) is an excellent modality for evaluation of ocular melanoma and to differentiate it from with other entities that may have similar clinical presentation. Both A-scan and B-scan ultrasonographies are useful in the evaluation of uveal melanoma. The classical appearance of choroidal melanoma on B scans is a biconvex echogenic mass (Fig. 1a). The tumours that have ruptured through Bruch’s membrane show a collar button configuration [7]. Continuous expansion of the tumour leads to exudative retinal detachment, subretinal haemorrhage and vitreous haemorrhage. Ultrasonography is also useful for detecting scleral invasion and trans-scleral extension of the tumour. Many ocular lesions such as choroidal nevus, choroidal metastasis, choroidal haemangioma, posterior scleritis and age-related macular degeneration (AMD) can resemble uveal melanoma on imaging and should be considered in the differential diagnosis.

**Fig. 2:**
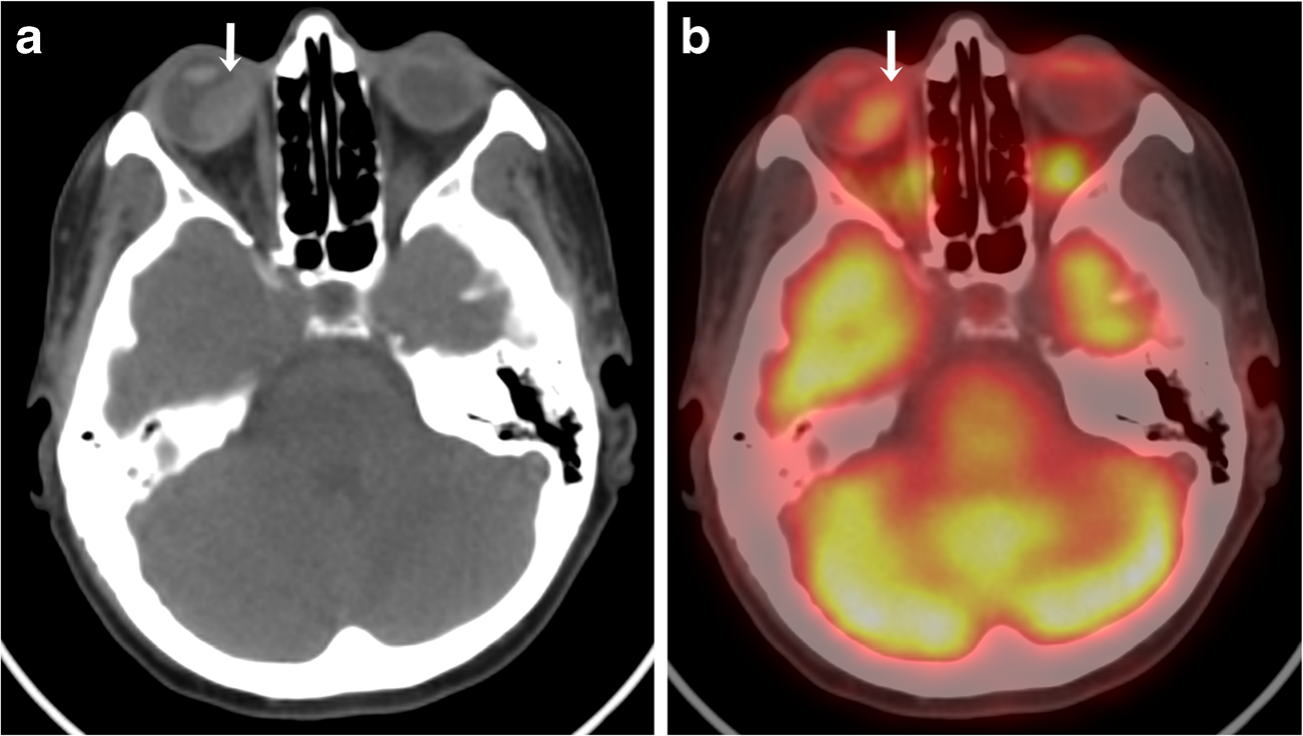
A 62-year-old male with choroidal melanoma. **a** Axial CT image shows a hyperdense mass in the posterior segment of the right globe (arrow). **b** The lesion shows moderate FDG uptake on the corresponding axial fused FDG-PET/CT image (arrow)

**Fig. 3:**
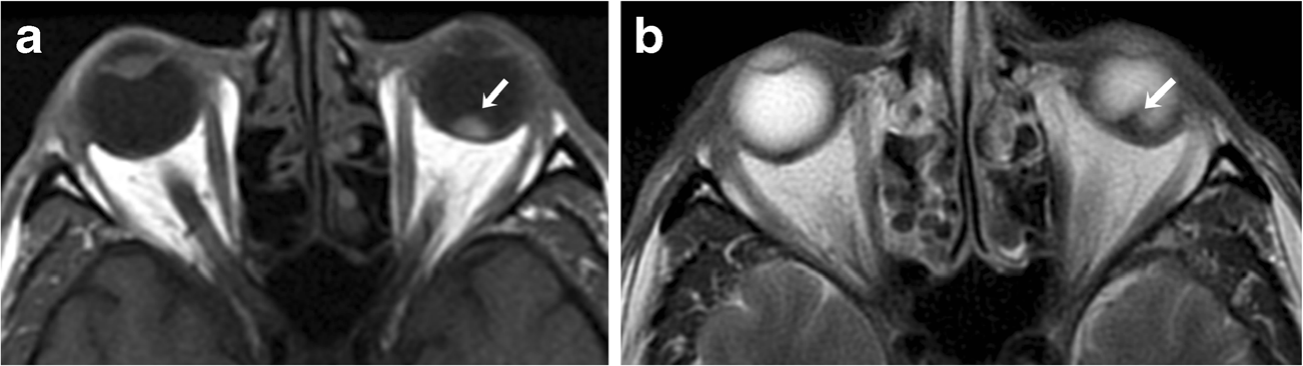
A 46-year-old male with uveal melanoma. **a** On the axial T1-weighted MR image of the brain, there is a T1 hyperintense choroidal mass (arrow). **b** The mass is hypointense on the corresponding axial T2-weighted MR image (arrow)

**Fig. 4:**
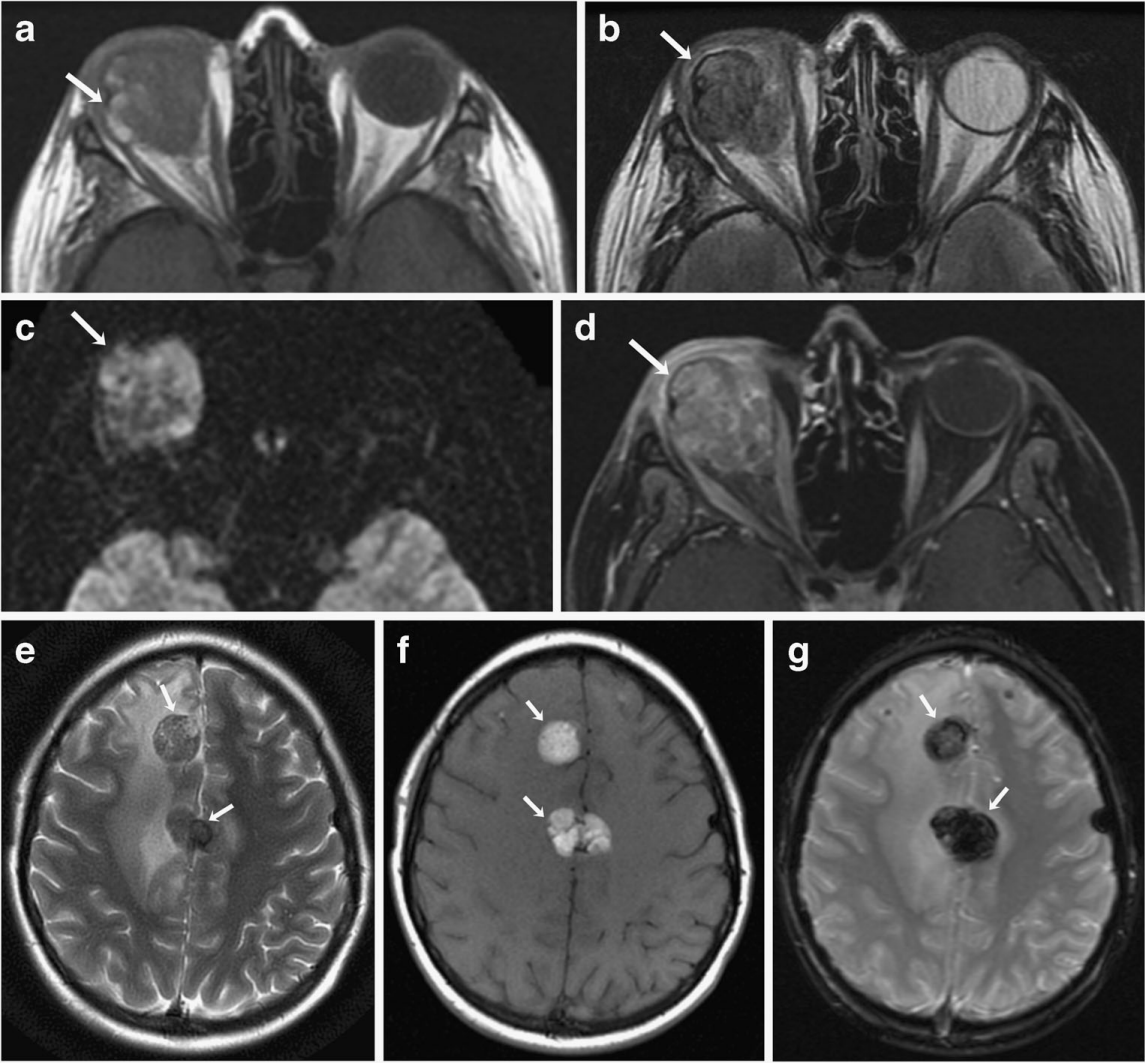
A 26-year-old male with ocular melanoma with extraocular extension and intracranial metastases. **a** Axial T1-weighted MR image showing a heterogeneous mass involving the right orbit with areas of T1 hyperintensity (arrow) and extraocular extension. **b** The mass is heterogeneously hypointense on the corresponding axial T2-weighted MR image (arrow). **c** On corresponding axial diffusion-weighted MRI, the mass shows restricted diffusion (arrow). **d** Axial contrast-enhanced T1-weighted MR image shows heterogeneous enhancement of the lesion (arrow). **e** Axial T2-weighted MR image shows metastatic lesions in the frontal lobes showing T2 hypointensity (arrows) with surrounding vasogenic edema. **f** The lesions are hyperintense on the corresponding axial T1-weighted MR image of the brain (arrows). **g** On the corresponding axial susceptibility-weighted (SW) MRI the lesions are hypointense (arrows) confirming a haemorrhagic component

CT protocol for orbital imaging consists of thin-slice reconstruction in both soft tissue and high-resolution bone algorithms. On CT, uveal melanoma appears as a hyperdense lenticular or mushroom-shaped lesion with post-contrast enhancement (Fig. 2). The MRI protocol for orbits includes T2 STIR (short tau inversion recovery), T1 and T1 fat-saturated TSE (turbo spin echo) sequences with 3-mm slice thickness and post-contrast fat-saturated T1 images in axial, coronal and oblique sagittal planes. On MRI, these lesions typically show characteristic T1 hyperintensity and T2 hypointensity due to paramagnetic effects of melanin, which causes T1 and T2 shortening (Fig. 3a and b). However, mildly pigmented lesions and amelanotic melanomas may be isointense to the vitreous body on T1-weighted images [9]. MRI is particularly useful for evaluation of optic nerve and retrobular soft tissue because of its superior contrast resolution (Fig. 4a-d). High-resolution MR imaging with surface coils is accurate for evaluating potential extrascleral extension of uveal melanoma, which can change the management of these tumours [10].

Orbital melanoma is generally first detected on opthalmoscopic examination. US is usually the first imaging modality used for further evaluation melanoma and is helpful in differentiating it from ocular pathologies. Cross-sectional imaging modalities such as CT and particularly MRI are useful for the exact delineation of the tumour and evaluation of optic nerve involvement and extraocular extension. PET/CT is useful for evaluation of metastatic disease, providing a whole-body evaluation in one session and also for assessment of treatment response.

#### Primary malignant melanoma of the CNS

Primary melanoma of the central nervous system is a rare tumour that accounts for approximately 10 % cases of CNS melanoma, the rest being metastases to the CNS from other primary sites [11]. Primary CNS melanoma arises directly from melanocytes within the leptomeninges, derived from the neural crest during early embryonic development [12, 13]. Primary melanoma of the CNS may present with either localised intra-/extraaxial mass lesions or diffuse meningeal melanomatosis. The discrete mass lesions can be extraaxial, intracerebral, intraventricular or intramedullary in locations [11, 14]. Primary intraventricular malignant melanoma arises from arrested melanocytic cells in the choroid plexus.

**Fig. 5:**
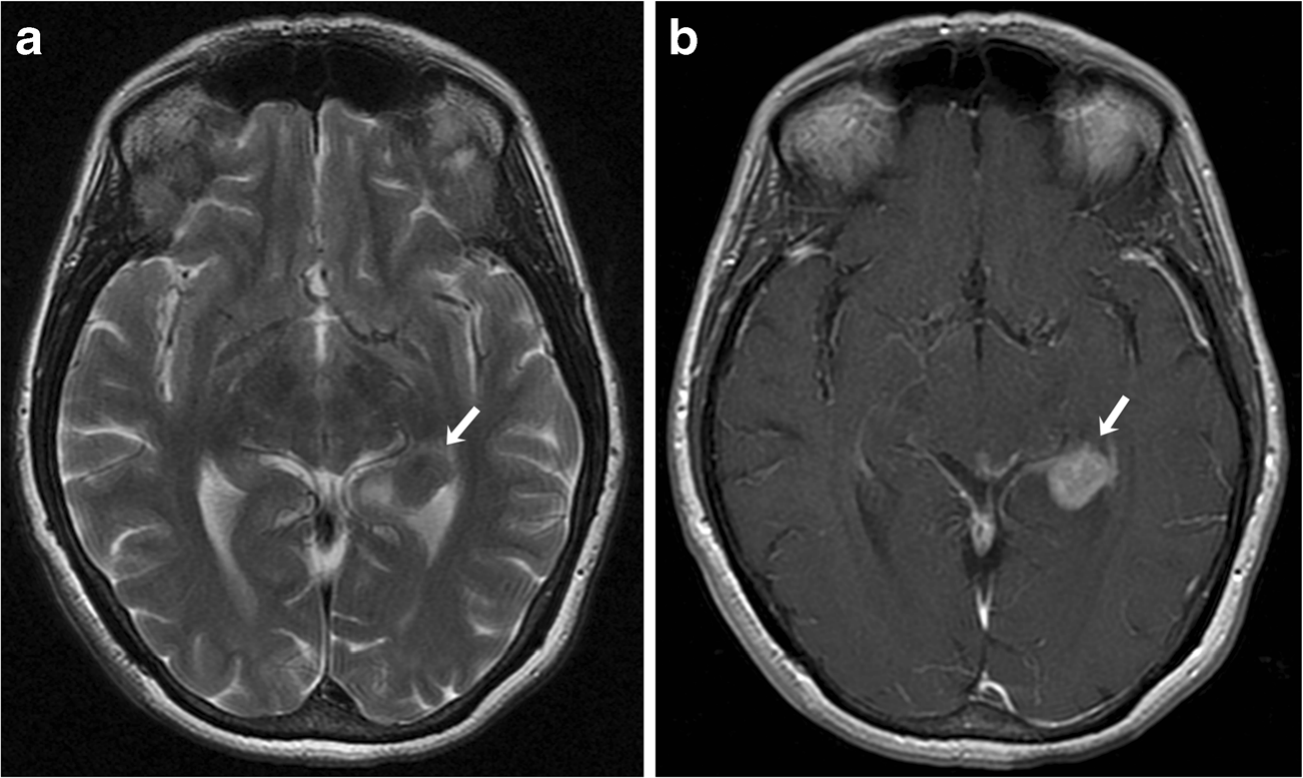
A 54-year-old female with choroidal melanoma. **a** Axial T2-weighted MR image showing an intraventricular mass lesion with T2 shortening involving the left lateral ventricle (arrow). **b** On the corresponding axial contrast-enhanced T1-weighted MR image, the lesion shows avid enhancement (arrow)

On imaging, primary CNS melanoma is indistinguishable from metastatic melanoma and is usually a diagnosis of exclusion after ruling out primary sites involving the skin, eyes and mucosal surfaces. The lesions are generally hyperdense on CT and show T1 hyperintense and T2 iso-hypointense signal on MRI with variable enhancement on post-contrast sequences (Fig. 5). [15]. Localised leptomeningeal melanoma can mimic meningiomas and acoustic neuromas, with definitive diagnosis made only on histopathological examination. Primary diffuse meningeal melanomatosis shows diffuse dural leptomeningeal thickening and enhancement on CT/MRI [16].

### Mucosal melanoma

#### Epidemiology

Mucosal melanoma arises from the mucosal epithelium of the respiratory, gastrointestinal and genitourinary tracts and accounts for 1 to 2 % of melanoma [17]. Mucosal melanoma usually occurs in middle-aged and elderly patients with a median age of 70 years. Approximately 30 % of mucosal melanomas are multifocal [18]. The head and neck region is the most common site of mucosal melanoma in males and the second most common site in females, accounting for approximately 25 to 50 % of all cases of mucosal melanoma [1]. The incidence rate for mucosal melanoma arising from the genital tract, rectum and anal canal is higher in females compared to males.

#### Clinical features, staging and diagnostic workup

Mucosal melanomas include head and neck, vulvovaginal, anorectal and urinary tract melanomas. More than 50 % of cases of mucosal melanoma arise from head and neck mucosal surfaces. The nasal cavity is the most common head and neck site for mucosal melanoma followed by the oral cavity [1]. Less frequent sites in the head and neck region include the pharynx, larynx and oesophagus. Clinically mucosal melanomas arising from the nasal cavity and paranasal sinuses present with nasal obstruction, epistaxis and anosmia [19]. Oral mucosal melanoma is frequently detected as mucosal pigmentation, bleeding or ulceration [20]. The genital tract is the most common site of mucosal melanoma in females, responsible for more than 50 % cases [1]. In females, the vulva is the most common site for genitourinary melanoma, followed by the vagina. Anorectal mucosal melanoma represents approximately 0.05 percent of all colorectal malignancies [21]. The rectum is the most common site (42 %), followed by the anal canal (33 %) and overlapping areas (25 %) [21]. HIV infection increases the risk of anorectal melanoma [22].

In 2010, the AJCC developed a specific TNM staging system for head and neck mucosal melanoma. According to the AJCC, staging of mucosal melanomas of the head and neck is done according to the tumour, node and metastasis (TNM) classification with disease confined to the mucosa designated as T3 to reflect their aggressiveness (Table 1) [23]. Contrary to head and neck mucosal melanoma, there is no universal staging system for anorectal and vaginal melanoma. The Ballantyne staging system, which was originally developed to stage head and neck melanomas, is generally used to stage anorectal and vaginal melanomas (Table 2) [2, 24, 25]. The AJCC-2002 staging system for cutaneous malignant melanoma is used to stage vulvar melanoma and is the most significant predictor of recurrence­free survival (Table 3) [26]. The diagnostic workup for mucosal melanoma includes endoscopic examination and various imaging modalities including CT, MRI and PET/CT. CT and/or MRI are used for evaluation of the primary site of disease in various mucosal melanomas, for delineation of local extent of disease and regional lymph nodal involvement. MRI is particularly useful in differentiating melanoma from other tumours because of its high soft tissue resolution and ability to depict characteristic T1 hyperintensity and T2 hypointensity of melanin. Evaluation of lymph node involvement and metastatic disease is usually done by CT or PET/CT. CT and/or PET/CT is also used for assessment of response to treatment.

**Table 1:** TNM Classification of Mucosal Melanoma of the Head and Neck

**Primary tumour (T)** T3: Mucosal disease T4a: Moderately advanced disease. Tumour involving deep soft tissue, cartilage, bone or overlying skin T4b: Very advanced disease. Tumour involving brain, dura, skull base, lower cranial nerves (IX, X, XI,XII), masticator space, carotid artery, prevertebral space or mediastinal structures **Regional lymph nodes (N)** NX: Regional lymph nodes cannot be assessed N0: No regional lymph node metastases N1: Regional lymph node metastases present **Distant metastasis (M)** M0: No distant metastasis M1: Distant metastasis present

**Table 2:** Ballantyne staging system

Stage	Disease extent
**I**	**Clinically localised disease**
**II**	**Regional nodal involvement**
**III**	**Distant metastatic disease**

**Table 3:** (AJCC) TNM staging system of vulvar melanoma

**Primary tumour (T)** TX: Primary (main) tumour cannot be assessed T0: No evidence of primary tumour Tis: Melanoma in situ T1: The melanoma is less than or equal to 1.0 mm thick T2: The melanoma is between 1.01 and 2.0 mm thick T3: The melanoma is between 2.01 and 4.0 mm thick T4: The melanoma is thicker than 4.0 mm T1-T4 stages are subdivided into a (without ulceration) and b (with ulceration) **Regional lymph nodes (N)** NX: Regional lymph nodes cannot be assessed N0: No regional lymph node metastases N1: Spread to 1 nearby lymph node N2: Spread to 2 or 3 nearby lymph nodes, OR spread of melanoma to nearby skin (known as satellite tumours) or toward a nearby lymph node area (known as in-transit tumours) without reaching the lymph nodes N3: Spread to 4 or more lymph nodes, OR spread to lymph nodes that are clumped together, OR spread of melanoma to nearby skin (satellite tumours) or toward a lymph node area and into the lymph node(s) **Distant metastasis (M)** M0: No distant metastasis M1a: Metastasis to skin, subcutaneous tissue or lymph nodes in distant parts of the body, with a normal blood LDH level M1b: Metastasis to the lungs, normal LDH level M1c: Metastasis to other organs or distant spread to any site along with an elevated blood LDH level	

#### Imaging findings

##### Head and neck mucosal melanoma

Most frequent sites of head and neck mucosal melanoma are mucosa of nasal cavity, oral cavity and paranasal sinuses [27]. Among sinonasal melanoma the most common sites of origin are the nasal septum, lateral nasal wall, paranasal sinuses, and the inferior and middle turbinates. Within the oral cavity, the upper alveolus and hard palate are frequent sites. Sometimes the origin of the primary tumour is difficult to assess because of the large size and multifocal nature of the tumour. Lymph node metastases are seen in up to 25 % of patients at presentation, mostly involving the submandibular and upper jugular groups [28]. Lymph node metastases are less common in sinonasal melanoma in comparison with oral mucosal melanomas [27]. Adverse prognostic factors in head and neck mucosal melanomas are clinical stage at presentation, tumour thickness greater than 5 mm, vascular invasion and distant metastases [27].

**Fig. 6:**
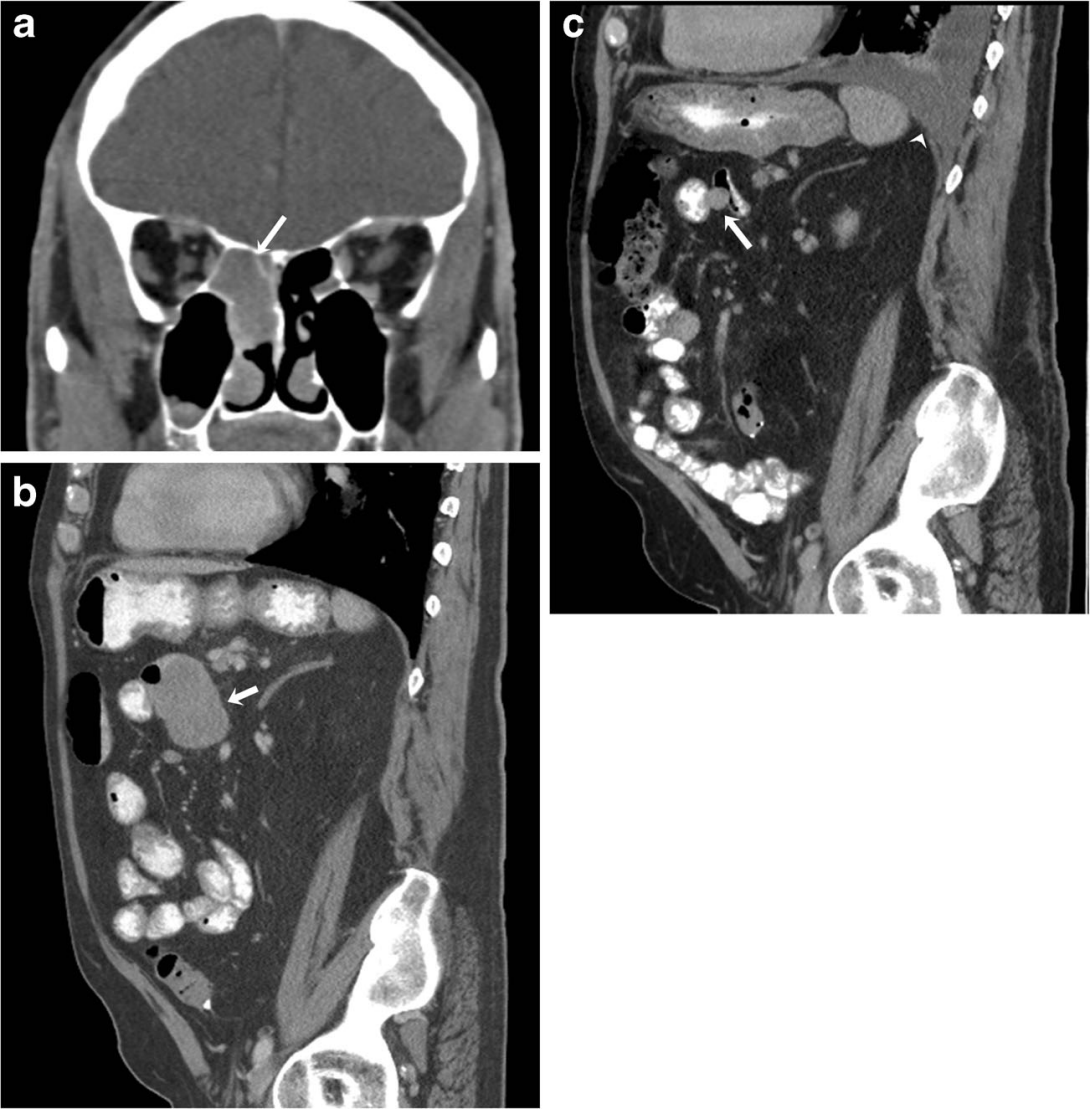
A 73-year-old male with metastatic sinonasal melanoma on treatment with BRAF and MEK inhibitors. **a** Coronal contrast-enhanced CT image shows a homogeneous mass lesion involving the right nasal cavity (arrow). **b** Sagittal contrast-enhanced CT image before the start of chemotherapy shows a large small bowel metastatic lesion (arrow). **c** Sagittal contrast-enhanced CT image after four cycles of chemotherapy shows a significant interval decrease in the size of the metastatic lesion and new left moderate pleural effusion (arrowhead)

The CT appearance of sinonasal melanoma is nonspecific and consists of a mass lesion with contrast enhancement and adjacent osseous destruction (Fig. 6a). The MRI appearance depends on the histological features of the lesion (melanotic verus amelanotic) and presence of haemorrhage within the lesion [29]. Melanotic melanomas typically exhibit T1 hyperintensity and an intermediate-hypointense pattern on T2-weighted MRI. Amelanotic melanomas are generally hypointense on T1- and hyperintense on T2-weighted sequences. The lesions demonstrate mild to moderate enhancement on gadolinium-enhanced MRI [29]. The differential diagnosis of T1 hyperintense lesions includes haemorrhage in primary nasal lesions such as haemangioma and juvenile angiofibroma, proteinaceous secretions in mucoceles, fat-containing lesions and haemorrhagic metastases.

##### Vulvovaginal melanoma

**Fig. 7:**
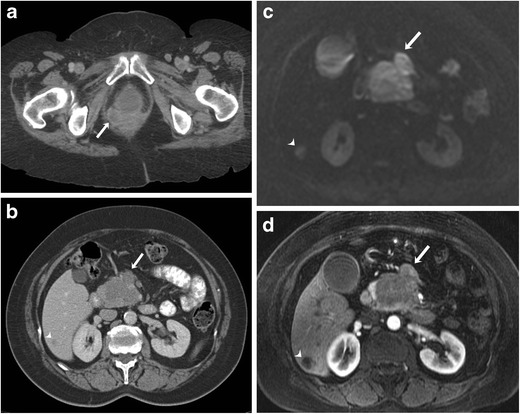
A 63-year-old female with vaginal melanoma and pancreatic and hepatic metastases. **a** Axial contrast-enhanced CT image shows heterogeneously enhancing vaginal mass (arrow). **b** Axial contrast-enhanced CT image shows a heterogeneous mass involving the pancreatic head (arrow) and an ill-defined hypointense lesion in the right lobe of the liver (arrowhead). **c** On corresponding axial diffusion-weighted MRI, the lesions show restricted diffusion. **d** Axial contrast-enhanced fat-suppressed T1-weighted MR image shows pancreatic (arrow) and hepatic (arrowhead) metastatic lesions

Vulvar melanoma is the most common site for genital melanoma accounting for 76 % of cases, followed by vaginal melanoma (20 %) [1]. Melanoma is the second most common vulvar malignancy after squamous cell carcinoma. CT findings in genital melanoma are nonspecific and consist of enhancing mass lesion(s) and invasion into surrounding organs (Fig. 7a). Genital melanoma shows iso- to hyperintense signal on T1-weighted MRI and usually hyperintense signal on T2-weighted images. Apart from conventional MRI sequences, dynamic contrast-enhanced MRI and diffusion-weighted imaging (DWI) are also useful in the evaluation of melanoma. On DWI, vaginal melanoma exhibits hyperintense signal with a low apparent diffusion coefficient (ADC) value. On dynamic post-contrast images, they show a rapid rise type curve with significant enhancement during the early phase due to the hypervascular nature of these tumours [30]. The differential diagnosis for genital melanoma includes epithelial as well as various non-epithelial tumours such as lymphoma, leiomyosarcoma and schwannoma.

##### Anorectal melanoma

**Fig. 8:**
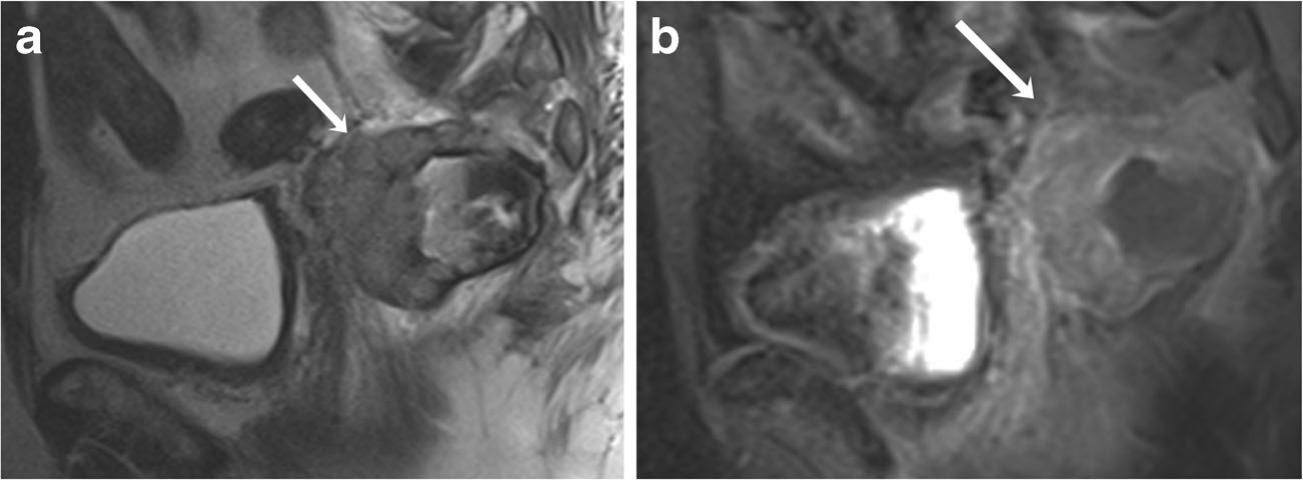
A 76-year-old male with anorectal melanoma. **a** Sagittal T2-weighted MR image shows a heterogeneous mass lesion arising from the anterior wall of the rectum (arrow). **b** The mass shows enhancement on the corresponding sagittal fat-suppressed T1-weighted MR image (arrow)

is usually seen as an intraluminal polypoid or fungating mass in the distal rectum or anal canal. Perirectal infiltration of the tumour is characterised by extension to the pelvic side wall and the pre-sacral space. Lymph node spread is commonly seen at presentation and occurs in the perirectal, internal iliac, obturator and inguinal groups. Luminal obstruction is less common in anorectal melanoma [31]. The main differential diagnosis for anorectal melanoma is adenocarcinoma, which is frequently seen as an obstructive mass because of infiltration and narrowing of the lumen. MRI is particularly useful for preoperative staging in anorectal melanoma patients, in particular to assess bowel wall invasion, because of its superior contrast resolution compared to CT and multiplanar imaging capacity. Similar to other sites, anorectal melanomas are hyperintense on T1-weighted images and show mixed signal intensity on T2-weighted images (Fig. 8a) with marked enhancement on post-contrast T1-weighted images (Fig. 8b); however, amelanotic melanomas, which comprise approximately 10 to 29 % of anorectal melanomas, are hypointense on T1- and hyperintense on T2-weighted sequences [32].

#### Metastatic disease

Up to 95 % of patients with mucosal melanoma have metastatic disease at presentation or develop it during the course of their disease [33]. Common sites of metastatic disease are the lung, liver, peritoneum and central nervous system. Less common sites of metastatic disease include the skin, subcutaneous tissues and bones. Approximately 50 % of all patients with primary uveal melanoma will develop metastatic disease [8]. The liver is the most commonly involved site of metastases in approximately 90 % of patients with ocular melanoma. Common extrahepatic sites of metastases are the lung, bone and skin [8].

#### Pattern of metastases in mucosal melanoma

**Fig. 9:**
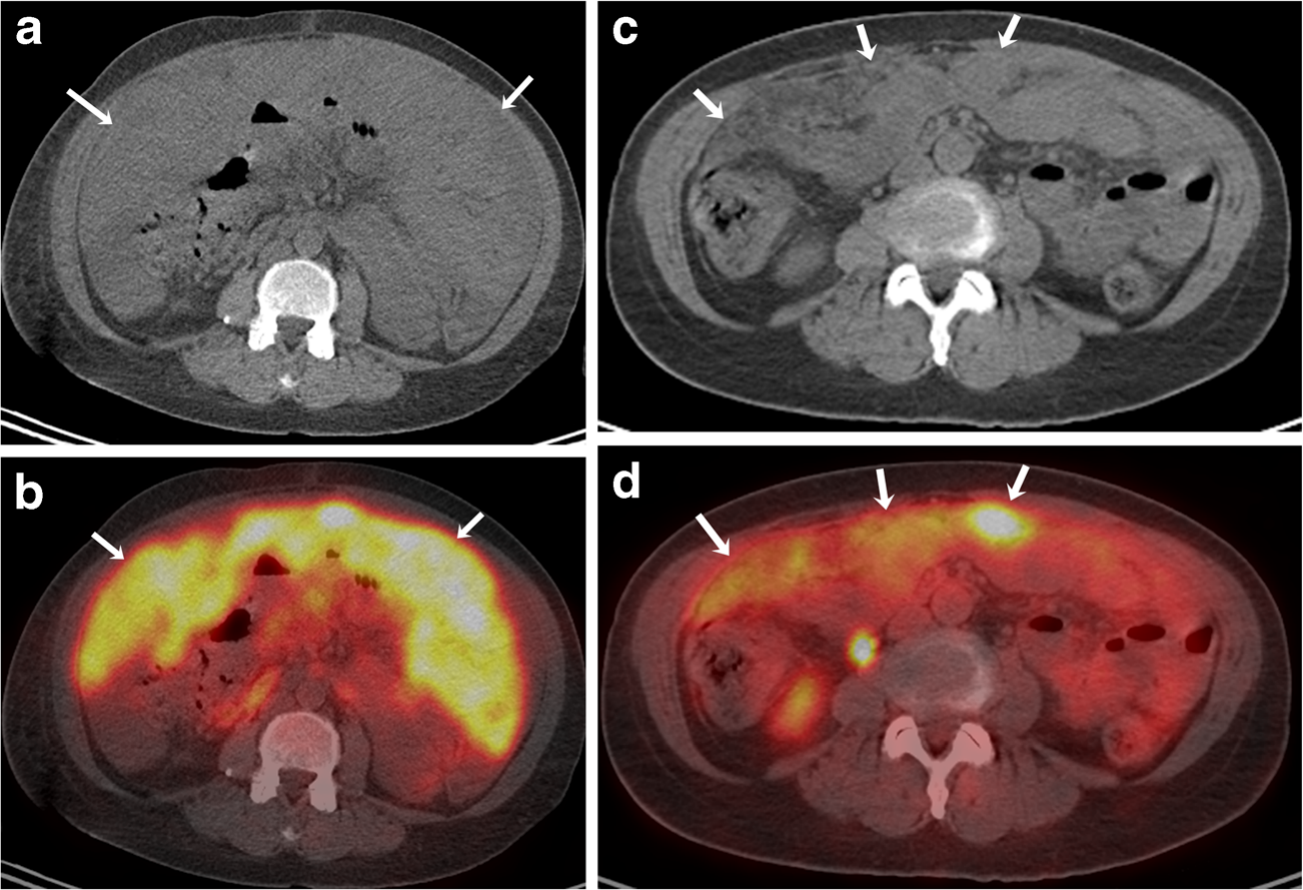
A 61-year-old female with vaginal melanoma and peritoneal metastasis. Axial unenhanced CT (**a**) and fused FDG-PET/CT (**b**) images show diffuse peritoneal thickening (arrows in **a**) with intense FDG uptake (arrows in **b**). Axial unenhanced CT (**c**) and fused FDG-PET/CT (**d**) images after treatment with imatinib shows a significant decrease in peritoneal thickening (arrows in **c**) and FDG uptake (arrows in **d**)

The pattern of metastases depends upon the primary site of mucosal melanoma. Vaginal melanoma has a predilection for early peritoneal tumour spread because of the close anatomic relationship of the vagina to the peritoneal surface (Fig. 9) [33]. In many patients with vulvovaginal melanoma, distant metastatic disease can develop even in the absence of regional lymph node involvement (Fig. 7b-d). Sinonasal melanomas commonly metastasise to the liver. Lymph node spread is seen in approximately 60 % cases of anorectal melanoma [28]. Anal melanoma commonly spreads to the inguinal lymph nodes because of the lymphatic drainage to that site [33]. Common sites of distant metastases for head and neck melanoma are the lung, liver, bone and brain.

#### Imaging in metastatic disease

Assessment of metastatic disease in extracutaneous melanoma is done with various imaging modalities including radiography, US, CT, MRI and PET/CT, each having its advantages and limitations. Contrast-enhanced MRI is the gold standard for evaluation of metastatic involvement of brain. The role of PET/CT for evaluation of brain metastases is somewhat limited because of background cerebral metabolic activity [34]. The typical appearance of metastatic intracerebral melanoma on MRI is lesions of high signal intensity on T1-weighted images and low signal intensity on T2-weighted images, seen in approximately 50 % patients, because of the effects of both free radicals in melanin as well as a haemorrhagic component (Fig. 4e-g) [35]. MRI is also the modality of choice for the detection and characterisation of metastatic liver lesions because of its higher contrast resolution and lack of ionising radiation (Fig. 1b and c). US is helpful for differentiating between solid and cystic liver lesions; however it has inferior sensitivity and specificity compared to CT or MR imaging. On CT, hepatic metastases are often hypervascular and usually show enhancement during the late arterial phase and become hypoattenuating to background liver parenchyma in the portal venous phase [36, 37] . CT is also the most sensitive imaging modality for pulmonary metastases [38]. PET/CT is becoming increasingly popular in the staging of metastatic extracutaneous melanoma. Because malignant cells usually have a higher metabolic rate, they show greater FDG avidity than adjacent normal tissues. PET-CT often helps in treatment planning based on the stage of disease as it provides evaluation of various metastasis sites in a single examination (Fig. 9a-d).

Prognosis of mucosal melanoma depends upon the primary site and stage of the disease, with melanomas of the oral cavity and external genitalia having a better prognosis as compared to other mucosal locations [17]. The prognosis of pharyngeal, gastroesophageal and vaginal melanoma is usually poor. The long-term prognosis of anorectal melanoma is poor, with overall 5-year survival of about 15 to 20 %, even in patients with surgically resectable disease at presentation [2]. Lymph nodal involvement and presence of distant metastases at presentation are adverse prognostic factors and associated with poor overall survival. Surgery is the primary treatment for mucosal melanomas if the lesion can be resected with negative margins; however, complete surgical resection with adequate margins is often not possible. In these cases and advanced disease, systemic treatment becomes important [39, 40].

### Systemic treatment for advanced stage extra-cutaneous melanoma and recent treatment advances

Systemic treatment of patients with metastatic melanoma arising from mucosal and uveal primaries is largely based on the experience on systemic treatment of metastatic cutaneous melanoma. Commonly used cytotoxic drugs in patients with metastatic melanoma include dacarbazine, temozolamide, interleukin-2 and paclitaxel. In the past few years, major advances have been made in the molecular understanding of melanoma with identification of multiple oncogenes and development of small molecular targeted therapies against them [41].

BRAF is the most commonly mutated oncogene in malignant melanoma, and the BRAF mutation is seen in approximately 50 % of cutaneous melanomas [42]. BRAF mutations are seen in about 10 % of mucosal melanomas. Vemurafenib and dabrafenib are inhibitors of mutated BRAF signaling. Trametinib is an orally active small molecule inhibitor of MEK1 and MEK2 in the mitogen-activated protein kinase (MAPK) signal transduction pathway. Sequential inhibition of the MAPK pathway by combination therapy with dabrafenib (a selective BRAF inhibitor) and trametinib (MEK inhibitor) has been shown to improve the progression-free survival in patients with BRAF V600 melanoma (Fig. 6b and c) [43]. The US Food and Drug Administration (FDA) has approved drugs for BRAF mutant melanoma including the BRAF inhibitors vemurafenib (August 2011) and dabrafenib (May 2013) as well as the MEK inhibitor trametinib (May 2013).

Uveal melanoma has a different molecular pathogenesis compared with cutaneous and mucosal melanoma. Mutations in BRAF, KIT and NRAS are rarely seen in uveal melanoma; however more than 80 percent of uveal melanomas have mutations in GNAQ or GNA11. The GNAQ and GNA11 mutations lead to the activation of the downstream MAPK pathway, which may be a potential target for therapeutic intervention [44]. According to a phase 2 clinical trial in patients with metastatic uveal melanoma, treatment with selumetinib (a selective inhibitor of MEK1 and MEK2) resulted in a modestly improved progression-free survival and response rate compared with chemotherapy [45]. Mucosal melanomas have an increased prevalence of c-KIT mutations, seen in about 20-25 % cases [41]. Imatinib is a small molecule inhibitor of KIT that inhibits proliferation and induces apoptosis in melanoma cells harbouring KIT mutations, associated with significant clinical response in these patients [46].

**Fig. 10:**
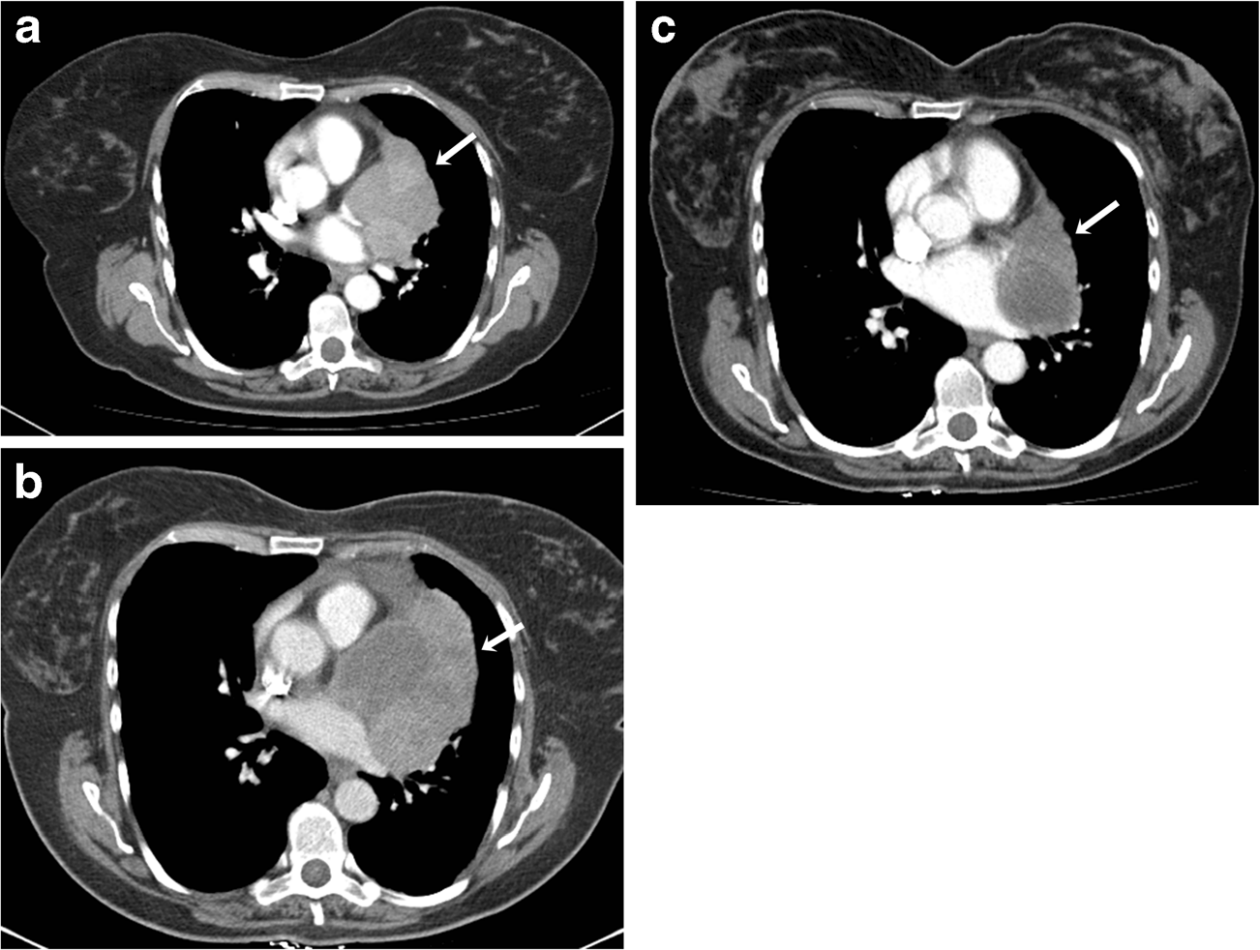
A 57-year-old female with metastatic melanoma from anorectal primary on treatment with ipilimumab. **a** Axial contrast-enhanced CT image before the start of chemotherapy shows a large heterogeneous paracardiac mediastinal mass (arrow). **b** Axial contrast-enhanced CT image 1 month after the start of treatment shows an increase in the size of the mass, however with a decrease in enhancement (arrow). **c** Axial contrast-enhanced CT image 1 month after completion of treatment shows a decrease in the size and enhancement of the mass (arrow)

Ipilimumab is a monoclonal antibody that was approved for the treatment of metastatic cutaneous melanoma by the FDA in March 2011. In recent clinical trials, ipilimumab has also been found useful in patients with metastatic uveal and mucosal melanomas [25, 47]. Ipilimumab activates the immune system by targeting cytotoxic T-lymphocyte-associated protein 4 (CTLA-4). CTLA-4 is a key downregulator of the immune system and blockade of CTLA-4 potentiates the T cell-mediated anti-tumour immune response [48]. Inhibition of CTLA-4 by ipilimumab can lead to unique immune-related adverse events (irAEs) such as a rash, colitis, hepatitis, hypophysitis, thyroiditis and pancreatitis [49]. Because of the distinct biologic mechanism of immunotherapeutic agents, immune-related response criteria (irRC) have been developed to characterise additional recognised patterns of treatment response. There are four distinct response patterns associated with favorable survival: (1) shrinkage in baseline lesions, without new lesions; (2) durable stable disease; (3) response after an initial increase in total tumour burden; (4) response in the presence of new lesions (Fig. 10). Detailed discussion about organ-specific irAEs and irRC is beyond the scope of this article [50]. Pembrolizumab and nivolumab are newer generation immunotherapy drugs, which were recently granted accelerated FDA approval in September and December 2014, respectively. Pembrolizumab and nivolumab have higher response rates and less toxicity compared to ipilimumab.

## Conclusion

In conclusion, extracutaneous melanomas are clinically and biologically distinct from cutaneous melanomas because of the higher frequency of metastatic disease and poorer overall prognosis. Complete surgical excision is the treatment of choice whenever possible; systemic therapy in the form of conventional chemotherapeutic agents as well as novel targeted agents is used for advanced/metastatic disease. Multiple imaging modalities including US, CT, MRI and FDG-PET/CT play important roles in the evaluation of the primary tumour, assessment of metastatic disease and monitoring response to treatment. Radiologists should be aware of the typical imaging manifestations of extracutaneous melanoma, the distinct patterns of metastatic involvement, as well as treatment response and toxicities associated with newer molecular targeted and immunotherapies to optimally contribute to patient management.
